# Bringing immunofocusing into focus

**DOI:** 10.1038/s41541-023-00792-x

**Published:** 2024-01-09

**Authors:** Sriharshita Musunuri, Payton A. B. Weidenbacher, Peter S. Kim

**Affiliations:** 1 Stanford ChEM-H, Stanford University, Stanford, CA 94305 USA; 2 Department of Biochemistry, Stanford University, Stanford, CA 94305 USA; 3 Department of Chemistry, Stanford University, Stanford, CA 94305 USA; 4 Chan Zuckerberg Biohub, San Francisco, CA 94158 USA

**Keywords:** Vaccines, Immunology

## Abstract

Immunofocusing is a strategy to create immunogens that redirect humoral immune responses towards a targeted epitope and away from non-desirable epitopes. Immunofocusing methods often aim to develop “universal” vaccines that provide broad protection against highly variant viruses such as influenza virus, human immunodeficiency virus (HIV-1), and most recently, severe acute respiratory syndrome coronavirus (SARS-CoV-2). We use existing examples to illustrate five main immunofocusing strategies—cross-strain boosting, mosaic display, protein dissection, epitope scaffolding, and epitope masking. We also discuss obstacles for immunofocusing like immune imprinting. A thorough understanding, advancement, and application of the methods we outline here will enable the design of high-resolution vaccines that protect against future viral outbreaks.

## Introduction

Immunofocusing is a strategy to create immunogens that preferentially direct the humoral immune response towards a particular epitope of a protein and has emerged as a promising approach to developing vaccine candidates that can provide broad protection against elusive viruses.

The motivation for immunofocusing can be illustrated with known monoclonal antibodies (mAbs). There are multiple examples where passive transfer of mAbs has been shown to provide protection against viral disease, and in some cases to provide broad-spectrum protection against multiple viral strains, such as nirsevimab for RSV and VRC01 for HIV^1–3^. These provide rationale for developing vaccines that could elicit a neutralizing antibody response mimicking the protective mAb^4,5^. However, attempts to redirect the immune response towards the epitope targeted by a known mAb have not yet been shown to produce broadly effective vaccines against any virus in a clinical setting^6,7^.

This lack of success can be attributed in large part to a phenomenon called immunodominance. Immunodominance describes the heightened prevalence of antibodies directed towards specific epitopes; the resulting immune pressure drives positive selection of strain-specific antibodies against these epitopes^8^. While immunodominance is not well-understood, it is thought to be influenced by several factors including epitope accessibility and flexibility^9^, affinity/avidity of the initial B cell-antigen or peptide-MHC interaction that mediates T cell help, germline B cell precursor frequency^10^, and clonal deletion of autoreactive Abs^11–13^. Consistent with these notions, the evolution of broadly-neutralizing antibodies (bnAbs) often utilizes germline alleles that are rare in humans and requires extensive somatic hypermutation (SHM) as compared to typical strain-specific antibodies^14^. bnAbs also frequently target regions of viral proteins that tend to be less accessible or that elicit lower-affinity antibodies, making it challenging to reliably elicit bnAbs with vaccination^15,16^.

Specifically for influenza virus hemagglutinin (HA), the major glycoprotein responsible for binding and fusion to host cells, most bnAbs are known to target the stem region^17,18^, which is highly conserved but induces fewer B cells than the exposed, variable, immunodominant head region^19,20^, which elicits the primary strain-specific response. Historic challenges associated with universal vaccine development for influenza virus have been reviewed comprehensively by others^21^. Additionally, while much attention in the literature is given to bnAbs against HIV-1 Env, these antibodies are very rare; the overwhelming majority of antibodies elicited by vaccination or HIV-1 infection are against immunodominant, strain-specific Env epitopes^22–24^.

First discussed over 25 years ago, immunofocusing seeks to address these challenges to developing protective antibodies^25^. The essential concept of immunofocusing is to diminish B cell responses against off-target, non-neutralizing, subtype-specific, or other undesirable immunodominant epitopes, and towards a specific desirable target epitope^25–27^. The primary goal of immunofocusing is to redirect the immune response towards epitopes defined by mAbs, and away from immunodominant, non-productive responses, including the elicitation of non-neutralizing off-target antibodies that may cause antibody-dependent enhancement (ADE) of disease, or epitope-dependent autoimmune reactions^28,29^. The role of T cells, which are also involved in broadening immunity, has been discussed in detail elsewhere^30–32^.

Using protein engineering and unique delivery strategies to alter the exposure of desired mAb epitopes will be critical in approaching this problem and developing vaccine candidates. A major consideration for these approaches is the ability to generate vaccine candidates with differing levels of exposure of the desired epitope(s) on the protein, relative to the size and immunostimulatory potential of all exposed epitope(s)- referred to here as immunofocusing resolution. A lower-resolution vaccine exposes more epitopes outside that of the target epitope(s) or masks the off-target epitopes to a lesser degree, while a higher-resolution vaccine exposes fewer off-target epitopes and more efficiently masks them. Masking approaches aim to decrease the immunogenicity of specific epitopes, through modifications such as glycosylation. Given that bnAbs and non-neutralizing antibodies can share overlapping epitopes^33,34^, resolution is a critical consideration for the design of immunofocused vaccines that are broadly protective. We postulate that the ideal universal vaccine would employ the highest-resolution possible, exposing only the footprint of a neutralizing mAb that targets an evolutionarily conserved epitope in its native state.

**Fig. 1:**
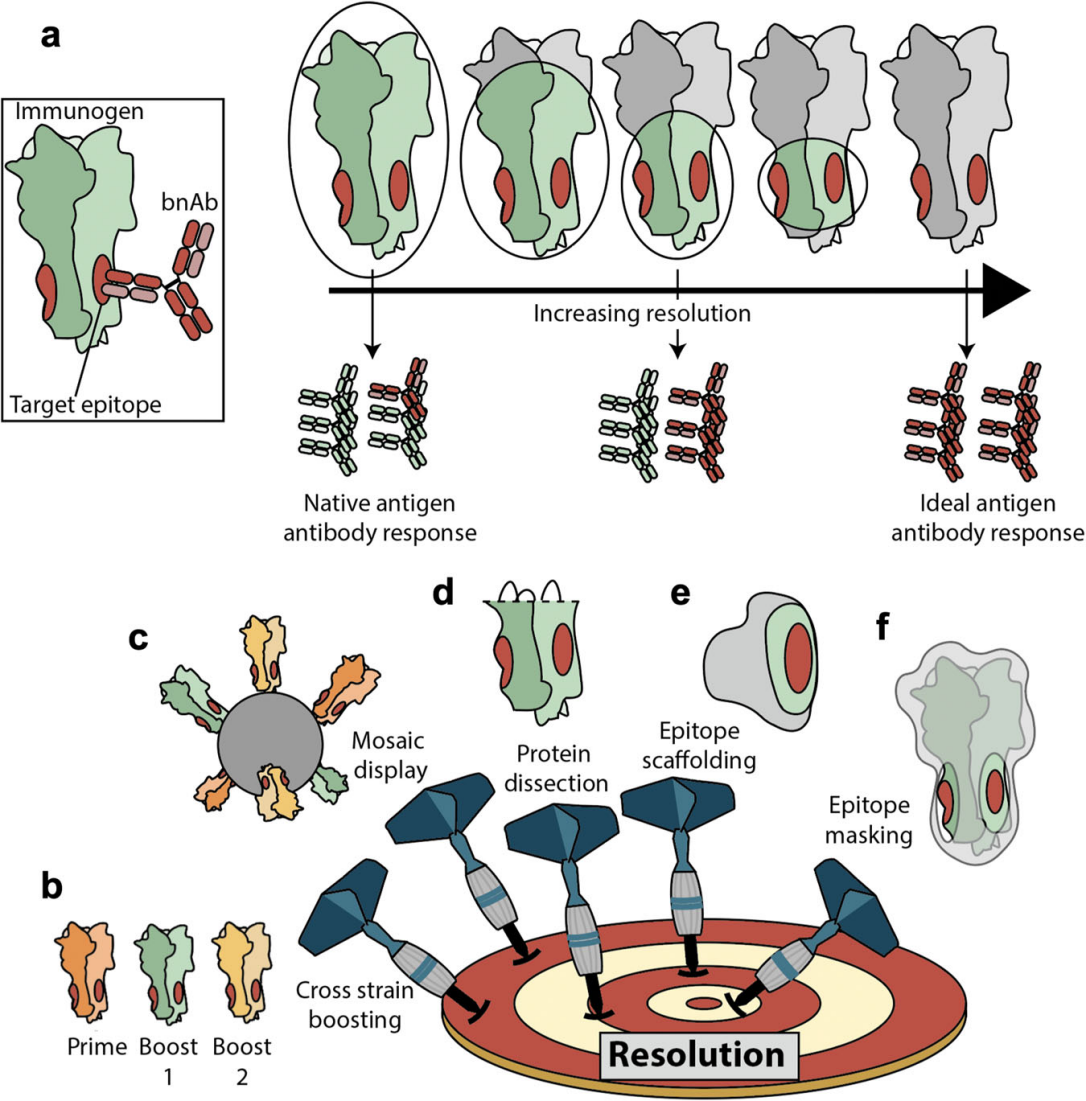
High-resolution immunofocusing. **a** The ideal broadly-protective immunogen elicits high titers of antibodies directed against the epitope of interest while avoiding antibody responses against off-target immunodominant epitopes. In the context of highly mutable viruses, this target epitope is defined by the binding footprint of a broadly neutralizing antibody (shown in red). Green represents off-target, exposed antigenic surfaces that are still capable of eliciting antibodies, while gray represents masked regions. **b**–**f** Five major classes of immunofocusing strategies: cross-strain boosting, mosaic display, protein dissection, epitope scaffolding, and epitope masking.

In this review, we discuss five primary methods of immunofocusing with varying resolution: cross-strain boosting, mosaic display, protein dissection, epitope scaffolding, and epitope masking (Fig. 1). We have selected a handful of studies that illustrate the strengths and shortcomings of each method. Our review complements several concepts that are well-covered by the recent review by Caradonna & Schmidt^35^, including multimeric display, stabilization of the prefusion conformation of viral immunogens, and germline-targeting antigens. Rather, we highlight the concepts of resolution, scaffold immunity, the distinction between free and antibody-bound epitope conformations in engineered immunogens, as well as control experiments to guide iterative vaccine design that should be considered when adopting immunofocusing methods.

## A note on original antigenic sin

An individual’s first exposure to a virus or immunogen shapes the responses to subsequent exposures^36^, a phenomenon known as immune imprinting or original antigenic sin (OAS)^37^. OAS is particularly well characterized for influenza virus^38,39^. Molecular fate mapping techniques to quantify OAS show suppression of de novo antibody responses by existing immunity that varies as a function of antigenic distance between the strains used to prime and boost^40^.

The ability to develop immunofocusing vaccines that are generally efficacious will need to account for, and in some cases overcome, preexisting immunity^41^. Oftentimes immunofocused antigens are tested in animal models that are näive to the pathogen of interest, which is not representative of previous exposures in humans that may skew the immune response. As well, new insights into germinal center dynamics in humans that seek to define mechanisms of immunodominance should inform childhood versus adult immunization schedules of immunofocusing vaccines as they advance clinically^42,43^. For instance, antibodies produced upon vaccination against the 2009 pandemic H1N1 showed a strain-specific response bias towards the immunodominant head, and limited development of a vaccine-induced response to stem epitopes in individuals with high pre-existing antibody levels, while low pre-existing antibody levels against the vaccinating strain was correlated with a stem-directed response^44^. Currently, infants are simply immunized with the latest influenza vaccine, with no regard for patterns of immunodominance. An immunofocused vaccine targeted to prime the development of stem-directed antibodies during infancy may prove fruitful towards shaping a long-lasting cross-reactive humoral response. Similar considerations have been discussed with regard to infant immunization with COVID-19 vaccines^45^.

## Cross-strain boosting

Cross-strain boosting involves sequential immunization with antigenically distinct versions of the same protein—with the aim of boosting cross-reactive B cells that outpace the strain-specific responses (Fig. 1b). Cross-strain boosting has been widely tested preclinically, but as with most immunofocusing methods, its primary applications have been against HIV-1 and influenza virus^46–48^, both of which are highly variable viruses with many different circulating strains. Early studies investigated sequential immunizations with different strains of disulfide-stabilized soluble gp140 trimers of HIV-1 Env (SOSIP), or with progressive boosts with zoonotic strains of influenza virus HA, and reported increased efficacy over single administration with multiple strains or sequential immunizations with the same strain^49,50^.

**Fig. 2:**
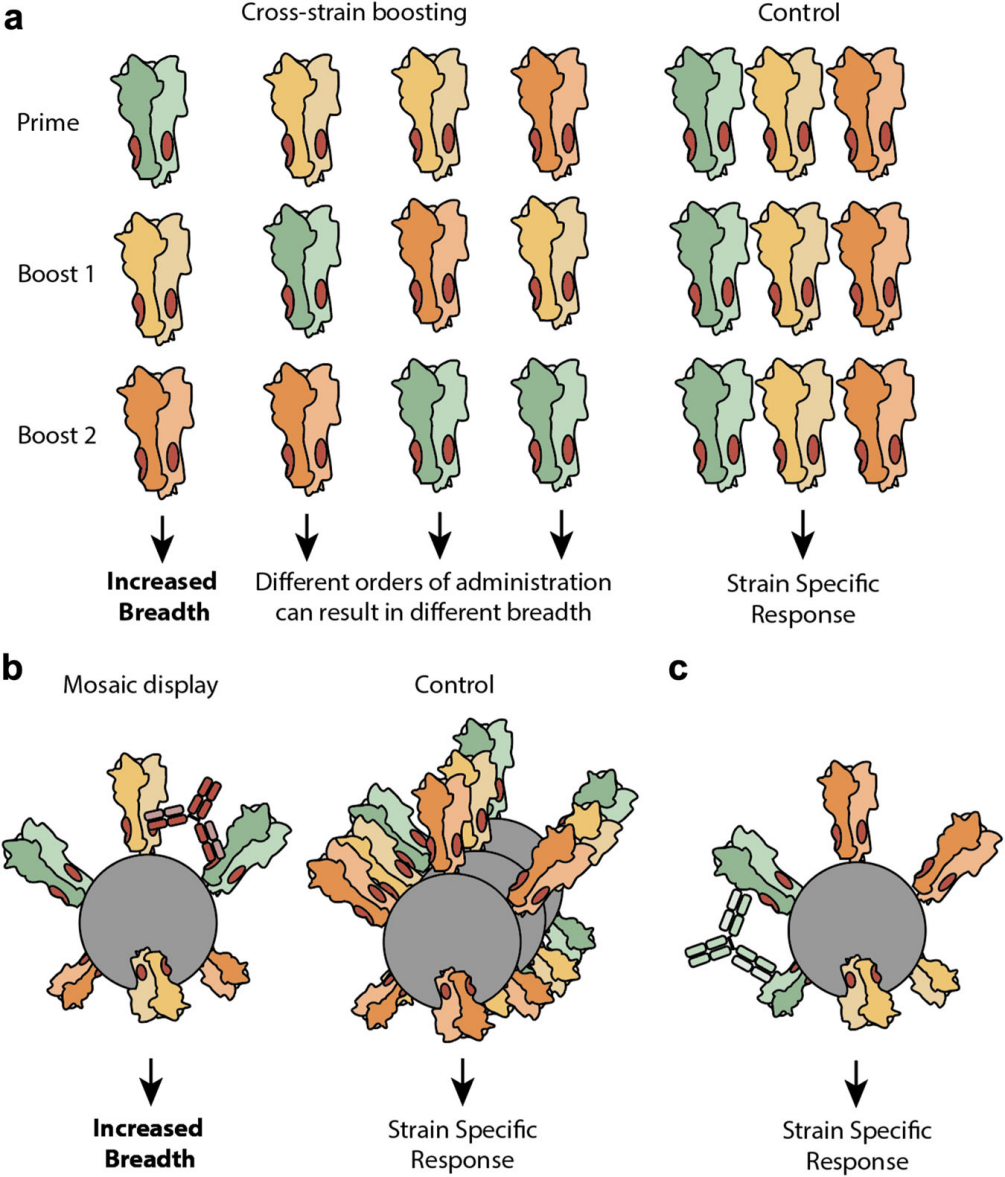
Cross-strain boosting and mosaic display. **a** In cross-strain boosting, antigenically distinct versions of the same protein are used for immunization. Varying the sequence in which antigen is administered can skew the antibody response and its breadth of neutralization, depending on which antigen is used as the prime^52^. To validate a multi-injection immunization schedule, cross-strain boosting studies should include a control where a mixture of heterologous antigens (each color represents a different strain of virus) is administered alongside antigens administered sequentially. This deconvolutes a poly-clonal strain-specific response from a cross-reactive response. **b** It is also the case with cross-strain boosting that a control mixture of homologous nanoparticles should be compared against immunization with mosaic nanoparticles to demonstrate increased neutralization breadth. Multivalent antigen display ideally preferentially elicits high avidity antibodies that cross-link adjacent antigens against the target epitope. **c** Strain-specific antibodies that are stimulated by adjacent homologous antigens at the surface of the nanoparticle can theoretically be minimized by increasing the number of variants displayed. This remains to be proven in an experimental setting.

Another example from Luo et al. used cross-strain boosting of virus-like particles (VLPs) containing either group 1 HA (H1, H8, H13) or group 2 HA (H3, H4, H10) in a challenge model^51^. Sequential vaccination with distinct VLPs provided improved protection compared to immunizations with a mixture of VLPs. The mixture of VLPs is an important control for any study testing the success of cross-strain boosting to support the conclusion that there is preferential boosting of cross-reactive B cells, as opposed to individual strain-specific B cells against each variant (Fig. 2a).

Van Reeth et al., tested sequential immunization with two H3 HA subtypes that shared ~80% sequence identity—one from Europe (G08 strain) and one from North America (PA10)^50^. Sequential immunization again performed better than a mixture of the two, stimulating higher antibody titers against divergent viruses. Following up on this work, Chepkwony et al. investigated if immunogen order altered the overall response^52^. The authors exposed pigs to the H3N2 A/Nanchang/933/1995 strain and then vaccinated with G08 followed by PA10 H3N2 or vice versa. The latter yielded better responses than the inverse. These results support the long-standing hypothesis of immunological imprinting or OAS, where immunogen order dictates responses (Fig. 2a)^36^. Similarly in humans, initial administration of an H7N9 antigen elicits a response with a greater breadth of neutralization than when an H5N1 antigen is administered first^53^.

In a conceptually similar method, Krammer and colleagues constructed chimeric HAs where the stem domains remained constant, but the head domains were derived from different strains. The authors found in a Phase I study that immunization with chimeric HAs redirected antibodies away from the immunodominant head and towards the conserved stem^54^. Passive transfer of antiserum from patients primed with chimeric H5 and boosted with H8 resulted in protection in mice against challenge with a heterologous virus.

Such clinical trial data is critical to support the use of cross-strain boosting for other viruses such as SARS-CoV-2. However, because cross-strain boosting does not shield off-target responses, it may not be ideal in all contexts. For instance, Jegaskanda et al. showed in non-human primates (NHPs) that sequential administration of an H5N1 vaccine followed by a seasonal vaccine can increase the stem-domain-directed response but the overall response is still dominated by a head-domain-specific response^55^.

A practical concern for cross-strain boosting is the requirement for multiple injections of distinct antigens over time, each of which are likely to require separate approval for clinical use. This complicates vaccination strategies, especially in resource-poor settings. Studies to improve the viability of the strategy include utilizing slow-release gels or patches with differing controlled distribution kinetics to sequentially release multiple strains following only a single injection^56–58^.

## Mosaic display

A second approach to immunofocusing is mosaic display (Fig. 1c). Mosaic display positions antigenically distinct versions of a protein on the same multivalent scaffold. Unlike multivalent display of homologous components^59–62^, mosaic display aims to specifically stimulate cross-reactive B cells since they can simultaneously engage common, targeted regions of the heterologous antigens (Fig. 2c).

Kanekiyo et al. demonstrated this effect by producing multivalent nanoparticles displaying receptor binding domains (RBDs - monomeric head regions) from an array of historic HA proteins^63^. The mosaic nanoparticle showed improved activity over either sequential immunization of homotypic nanoparticles or immunization with a mixture of homotypic nanoparticles. Again, the latter control was critical; comparison between immunization with the mosaic and immunization with a mixture of homotypic nanoparticles demonstrates that the improved activity from the mosaic sample was due to the geometry of the mosaic and not due to its multivalency. The authors further discussed the importance of nanoparticle geometry—specifically that mosaics should have no adjacent homologous antigens that could promote a strain-specific response. Given that the nanoparticles in Kanekiyo et al. were made via random assembly, increased antigen density and diversity decreases the likelihood of adjacent homologous antigens (Fig. 2c). Follow-up work by Kanekiyo demonstrated that mosaic immunogens displaying the four HA trimers found within the licensed quadrivalent flu vaccine elicited greater protection against vaccine-mismatched and heterosubtypic group 1 and 2 viruses when compared with immunization of a cocktail of homotypic nanoparticles^64^.

Separately, utilizing full-length HA trimers, Cohen et al. found that mosaic nanoparticles did not elicit broader immunity than the mixture of homotypic particles^65^, again highlighting the importance of this control (Fig. 2b). A potential explanation for the difference in neutralization breadth between the Kanekiyo and Cohen studies comes from the nanoparticle geometry, as Kanekiyo used monomeric RBDs expressed as genetic fusions to ferritin, and Cohen used full-length HA trimers conjugated via SpyCatcher-SpyTag. The latter increases distance between neighboring antigens and results in low conjugation efficiency, potentially mitigating some of the B cell activation potency conferred by multimeric antigen display^66^.

The mosaic display approach holds particular promise in light of the finding that mosaic SARS-CoV-2 RBD nanoparticles developed by Cohen et al. and further validated by Fan et al. were shown to protect against challenge by diverse sarbecoviruses (SARS-CoV-1 and SARS-CoV-2), while homotypic RBD nanoparticles protected only against SARS-CoV-2 challenge^67,68^. Epitope mapping of serum from mosaic-immunized animals demonstrated targeting of conserved epitopes, confirming a plausible mechanism to overcome immunodominance of highly variable RBD regions^68^. However, neither study compares the performance of a cocktail of homotypic nanoparticles to the corresponding mosaic nanoparticle, and thus it remains unclear the extent to which mosaic display enhances the breadth of the immune response against sarbecovirus RBDs. These nanoparticles also failed to provide protection against the SARS-CoV-2 BA.1 variant (Omicron), suggesting that neutralization of animal sarbecoviruses is not predictive of protection against closely related human variants of SARS-CoV-2 given differences in selective pressure on the virus.

As with cross-strain boosting, mosaic display does not mask off-target epitopes. Therefore, it is possible for immunodominant, strain-specific epitopes to still overwhelm the response at the expense of Abs against the conserved target epitope. Considering for instance that mosaic molecules made from similar proteins may elicit off-target antibodies, while those made from highly-divergent proteins may be unable to stimulate cross-reactive germline B cells, identifying the optimal sequence variation, geometry and density of displayed immunogens will be critical^41,62^. This includes ongoing efforts to design multi-component nanoparticle scaffolds to enable finer control over heterogeneous antigen assembly^69^.

## Protein dissection

**Fig. 3:**
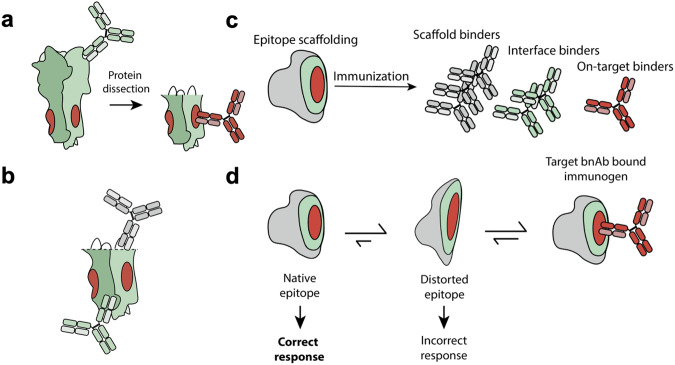
Protein dissection and epitope scaffolding. **a** Protein dissection can be an effective strategy to remove immunodominant regions and avoid undesirable antibody responses. **b** In protein dissection, native domains of the protein are replaced with non-native stretches of amino acids to stabilize the remaining structure. This can form neoepitopes that elicit undesirable antibody responses (grey) while leaving non-target regions of the native protein exposed (green). **c** Epitope-scaffolded immunogens can elicit antibodies directed towards the biologically irrelevant scaffold (grey) or the epitope-scaffold interface (green) that could skew the humoral response away from the desired epitope. **d** Confirming binding of the target Ab for the immunofocusing antigen is insufficient for characterizing how well it recapitulates the native antigen. This is because bnAb binding may shift the equilibrium of a distorted structure towards the native state. This distortion can be monitored by quantifying differences in affinity and structure.

A third immunofocusing method, protein dissection involves protein engineering to remove unwanted epitopes (Fig. 3a). It is the first method we discuss that actively discourages the elicitation of off-target antibodies, and is thus particularly useful for antigens with non-neutralizing immunodominant regions (Fig. 1d). Most studies that have applied protein dissection fall into two categories: 1) loop deletion: a long flexible loop within the protein is replaced with a shorter, more rigid structure, or 2) domain removal: an entire domain of the protein is removed, and the remaining structure is stabilized.

### Loop deletion

Loop deletion has primarily been applied to HIV-1, where flexible, hypervariable loops are thought to block neutralizing epitopes on Env, or where the loops themselves are immunodominant^70,71^. The rationale originates from early experiments demonstrating that removal of these loops increased affinity of neutralizing antibodies directed towards the CD4 binding site^72^. Several groups have explored the role of V1/V2 deletions on neutralization breadth and immunogenicity of HIV-1 vaccines^73,74^. However, these studies have not yet translated effectively to human studies. For example, a phase I immunization trial with trimeric gp140 with deletion of the V2 loop—a region identified to elicit high levels of non-neutralizing antibodies—demonstrated no improvement in neutralization breadth^75^. Loop deletion may remain a viable strategy for viruses with less stringent immunodominance hierarchies.

### Domain removal

While conceptually similar to loop deletion, removal of whole domains has a significantly larger impact on the antigenicity of the protein but may be necessary when deletion of only flexible loops is insufficient to avoid undesirable immune responses. This method has been explored in several different contexts including HIV-1^76^, RSV (head-only)^77^, and SARS-CoV-2 RBD^78^, among others, with varying success towards advancing to efficacy in patients.

Recent work by our lab showed that deletion of an immunodominant^79–81^, flexible, 70 amino acid region (which encompasses a large portion of the HR2 domain) from the C-terminus of the SARS-CoV-2 spike ectodomain results in an immunogen that elicits improved neutralizing activity compared to the full-length ectodomain - an effect that was particularly pronounced in multivalent immunogens^45,82^. Stabilized SARS-CoV-2 S2, which comprises the more conserved domain of the spike ectodomain, was also shown recently to elicit protective antibody responses in preclinical animal models^83^.

Unlike domains that are formed by a single stretch of amino acids (such as the RBD in the SARS-CoV-2 spike), some domains require more extensive engineering to stabilize the remaining structure. For example, through successive iterations of design, several groups have generated stabilized stem domains capable of mimicking the native stem by removing the variable head domain of influenza virus HA^84–88^. In one such case, short flexible loops were installed in place of the head domain, and the resulting H1 HA-stabilized stem was then multimerized onto a nanoparticle which displayed eight copies of the trimeric stabilized-stem, termed H1-ss-NP^85^. Passive transfer of serum from mice immunized with H1-ss-NP showed promise in a challenge model against heterologous H5N1, and this construct has now progressed into multiple phase 1 clinical trials^89,90^.

However, this particular stabilized stem construct faces several challenges including that the base of the stabilized stem trimer is splayed apart compared to the native structure, based on structures solved by cryo-EM and crystallography^85^. This seems to be responsible for differences in stem-directed mAb affinity for native HA and H1-ss-NP. Several groups developing headless HA immunogens have also identified a discrepancy between the robust cross-reactive binding and weaker, more subtype-specific, neutralizing activity of serum elicited by headless constructs^84,87^. Yassine et al. proposed that this results from the fact that the target bnAb epitope on the stem only constitutes about 15% of the surface area of the resultant stabilized stem^85^, such that a large portion of the response is likely directed at cross-reactive, but non-neutralizing epitopes. Additionally, domain removal may expose neoepitopes that are not representative of the native antigen (Fig. 3b). Thus, stabilized stem constructs may benefit from higher resolution immunogen engineering.

Another visible application of domain removal are the eOD-GT immunogens, which display the HIV-1 CD4-binding site (CD4bs) epitope on a minimal engineered outer domain^91^. Iterative mutations were made to create eOD-GT8, which binds with relatively high affinity to the CD4bs-directed bnAb VRC01 and its germline precursor. This was then multimerized onto a lumazine synthase nanoparticle to display 60 copies on a single particle to boost immunogenicity^91^. eOD-GT8 activates B cells displaying the purported VRC01 germline and was used to isolate precursor B cells from naive donors^92^. Preliminary phase 1 results suggest that this candidate may stimulate epitope-specific responses^93^.

However, eOD-GT8 demonstrates several obstacles that generalize more broadly to protein dissection, namely, that epitope context may play an important role in antibody-mediated neutralization. Many antibodies elicited by the eOD are directed to regions outside of the grafted CD4-binding site (CD4bs), and those that do recognize the CD4bs epitope bind to the native SOSIP trimers weakly^91^. Several possibilities explain this outcome: the angle of approach for the elicited antibodies is incompatible with the native trimer due to clashes with neighboring Env subunits, and/or the target epitope is not sufficiently immunogenic relative to the available off-target epitopes to elicit enough target-specific antibodies with detectable neutralization^94^.

## Epitope scaffolding

An alternative and theoretically higher-resolution method of immunofocusing is epitope scaffolding. Epitope scaffolding translocates the epitope of interest from its native context onto a different scaffold. This may involve transplanting the epitope into either (1) an existing but evolutionarily distant or orthogonal structural homolog, or (2) a novel designed scaffold. Scaffolding is easiest when a high-resolution structure of the epitope exists, and the epitope itself is rigid and easily delineated from the rest of the antigen (e.g., a contiguous stretch of amino acids rather than a complex conformational epitope).

Epitope scaffolding is particularly useful when immunofocusing towards transiently exposed epitopes that are not readily accessible on the native antigen^95,96^. For instance, peptide mimetics of the gp41 prehairpin intermediate enable stable display of an epitope that is transiently exposed upon transition from prefusion to the postfusion state of HIV-1 Env^97^.

### Structural homolog scaffolds

In general, identifying structural homologs for use as an alternative scaffold involves scanning structural databases for proteins with similar folds to the original epitope and has been done to target the HIV-1 membrane-proximal external region (MPER) and other HIV-1 epitopes^95,98–100^. For example, Stanfield et al. used the MPER-directed bnAb Z13e1 as a guide to identify interleukin 22 (IL-22) as an orthogonal structural homolog with a similar epitope fold to the antibody-bound structure^101^. The authors then grafted the MPER epitope onto the homologous region of IL-22 and showed Z13e1 Ab bound to the grafted protein. However, using isothermal calorimetry, the authors found that the interaction of Z13e1 with the epitope-scaffolded immunogen did not use a lock-and-key recognition mechanism, as is common for antibodies. Instead, it seemed to require a binding-induced conformational rearrangement, implying that the antibody binding was shifting the equilibrium to “pull” the protein into the correct conformation^102^. More explicitly, a high affinity Ab could be capable of binding a distorted epitope due to thermodynamic favorability. A distorted epitope, however, decreases the likelihood of eliciting the desired humoral response, as it fails to capture the native epitope that the host would encounter upon infection (Fig. 3d). Indeed, this construct failed to elicit a neutralizing response upon immunization.

Separate efforts to graft MPER epitopes have failed to elicit a neutralizing response as well^103^. This may be partly due to the intrinsic flexibility of the MPER epitope, which has been shown to adopt different conformations when bound to different bnAbs^104^. It is possible that flexible epitopes are poor candidates for epitope scaffolding. Studies have also shown that MPER-directed Abs rely more on lipid interactions, which are not included in the scaffolded epitope^105^, highlighting another key consideration—epitope context. Such interactions with the lipid bilayer or with glycans may be important for the antibody binding and may not be captured by a scaffolded epitope.

The Stanfield et al. work highlights another consideration: namely that bnAb binding to a vaccine candidate is not a sufficient indicator that the vaccine candidate displays the proper epitope conformation to elicit the bnAb (Fig. 3d). Indeed, this challenge is shared among all the immunofocusing methods we discuss here. Biophysical approaches to identify potential discrepancies between the free epitope and the antibody-bound epitope include isothermal calorimetry, X-ray crystallography/cryo-EM, biolayer interferometry, competition enzyme linked immunosorbent assays, and protein thermal melts (Table 1), since they allow the quantitation of how well an immunogen mimics the native epitope and can thus guide iterative development prior to immunization in animals.

**Table 1:** Biophysical characterization that aids in iterative immunogen design.

Biophysical Method	Purpose
**X-ray Crystallography**	Determine the exact positions and orientations of amino acids in the immunogen vs WT antigen when bound to the target Ab, especially at the paratope-epitope interface^15^.
**Biolayer Interferometry**	Quantify differences in binding affinity to WT antigen vs. the immunogen against the target antibody, as well as the off-target antibodies to verify immunofocusing^135^.
**Protein melts**	Measure melting temperatures of an immunogen compared to the native antigen to detect differences in thermostability that may be indicative of premature unfolding^142^.
**Cryo-EM**	Capture the epitope specificity of polyclonal sera elicited by an engineered immunogen in a high-throughput manner, specifically via methods such as Cryo-EMPEM^143^.
**Competition ELISA**	Precisely map preferential targeting of a given epitope by polyclonal sera by measuring immunogen binding in the absence or presence of competing Ab interactions^144^.
**Isothermal calorimetry**	Calculate differences in the theoretical and measured heat capacity of binding to determine if immunogen-Ab binding recapitulates WT epitope-Ab binding^101^.

Discrepancies in structure between free and antibody-bound immunogen may arise due to improper display of the target epitope in its native conformation. It is important to minimize this difference between the native and immunogen-displayed epitope structure prior to immunization. This can be done using biophysical characterization of the free immunogen, and assessing its ability to bind on-target antibodies^145^, which may inform construct redesign or mutagenesis to improve thermostability/rigidity.

Epitope scaffolding is poised for rapid advancement in the coming years. So far, it has been mainly limited by the requirement for structural knowledge gained from crystallography/cryo-EM structures. Tools such as AlphaFold and ESM-fold should accelerate the discovery of greater and more diverse scaffolds, as well as tools to generate novel sequences that fold into specific conformations^106,107^.

### De novo designed scaffolds

For some target epitopes, it may not be possible to identify ideal homologous structures to recapitulate the original epitope. In such instances, de novo design and computational protein folding techniques can be used to develop novel scaffolds that display the target epitope. Early reports of this approach suggest that it is more straightforward to graft a linear/continuous epitope^108,109^, but recent advances in computation as well as integrating design with directed evolution have enabled grafting of not only linear, but also discontinuous epitopes on to protein scaffolds^110–112^.

In the case of RSV, immunization with epitope-scaffold candidates based on the fusion glycoprotein epitope of the bnAb motavizumab elicited antibodies that were able to bind to the F protein of RSV but were unable to neutralize the virus^109^. This was not wholly unexpected, as the authors noted that the bnAb had a lower affinity for the scaffold compared to the full-length antigen by orders of magnitude, suggesting improper display of the native epitope.

## Epitope masking

The final, and theoretically highest-resolution immunofocusing method is epitope masking. Epitope masking uses biochemical modifications to shield off-target epitopes. Its high resolution arises because modifications can be made directly adjacent to, but not in, the target epitope. This approach often utilizes the full-length protein such that the structure of the target epitope is largely unperturbed, increasing the likelihood of eliciting bnAbs against the desired epitope. An example of epitope masking—antigen reorientation—was recently pioneered by our lab and others. We installed aluminum hydroxide (Alum) binding oligo-aspartate peptide loops onto the head domain of HA, thereby inverting it on the Alum surface to generate reorientated HA (reoHA)^113^. ReoHA elicits robustly cross-reactive and stem-specific responses compared to WT HA. An alternative approach used a streptavidin VLP to bind biotinylated HA in an inverted orientation^114^.

### Glycan repositioning

While epitope masking can be implemented in a variety of ways, the most common approach is glycan repositioning or, more specifically, hyperglycosylation^115^ via introduction of N-linked glycan consensus sequences^116^. Given their bulky, flexible nature, glycans are capable of sterically occluding epitopes. Indeed, viruses naturally utilize glycans to obscure epitopes and evade immunity^117^. Thus, the application of glycan repositioning is a natural choice for developing various potential vaccines, including for influenza virus^118,119^, HIV-1^120–123^, SARS-CoV-2^124^, and RSV^125^.

Early examples of glycan repositioning include the introduction of single glycan modifications that could redirect the immune response elicited by HA^118,119,126^, as well as the addition of N-linked glycans to dampen the immune response against the V3 loop on Env^70^. Subsequent adaptation of this approach produced a hyperglycosylated form of gp120 of HIV-1 Env, which retained binding to the neutralizing mAb b12 but reduced the binding of non-neutralizing antibodies^121^. This design was the result of iterative improvements to include additional glycosylation sites, and to remove a single glycosylation site and a fragment of the N-terminus. The resulting hyperglycosylated gp120 ultimately led to an overall dampening of the immune response, but did not improve the elicitation of b12-like antibodies^121^, again highlighting that in vitro antibody binding is not necessarily representative of in vivo elicitation of the humoral response.

Similar efforts to alter glycosylation profiles have been made by others to engineer HIV-1 immunogens^127^. Most of these studies applied iterative design informed by a panel of antibodies targeting on and off-target regions, a technique that is important not only for epitope-masked immunogens, but for evaluating the antigenicity of all immunofocused antigens.

### Antigen resurfacing

Antigen resurfacing introduces amino acid variations to regions outside of the target epitope with the goal of altering the immune profile of the antigen, ideally skewing the immune response towards the epitope of interest^25^. The first decisive example was applied to a model antigen, human chorionic gonadotropin (hCG), in which a single Arg to Glu substitution at position 68 shifted the resultant humoral immune response^128^. Resurfacing has since been applied to HIV, dengue, and other viruses with limited success^33,129^. While resurfacing modifications can alter the immune response of antigens, it is difficult to render these regions fully immunosilent.

Despite these setbacks, resurfacing remains an attractive method because of the simplicity of installing conservative, small substitutions, many of which can be accommodated on a single protein^130^. Future applications will likely take advantage of recent advances that have enabled our understanding of the entire sequence space and structural features of SARS-CoV-2 spike, HIV-1 Env, and other proteins by deep-scanning mutagenesis^131^.

### PEG modifications and PMD

Installation of polyethylene glycol (PEG) moieties is another form of epitope masking. Pioneering work by Davis and colleagues demonstrated many advantages of PEGylation for therapeutic applications, one of which is that PEGylated proteins are significantly less immunogenic than their non-PEGylated counterparts^132^. The first PEGylated therapeutic proteins, PEG-adenosine deaminase for adenosine deaminase deficiency^133^ and PEG-asparaginase for acute lymphoblastic leukemia^134^, support the notion that PEG can selectively dampen the immune response.

**Fig. 4:**
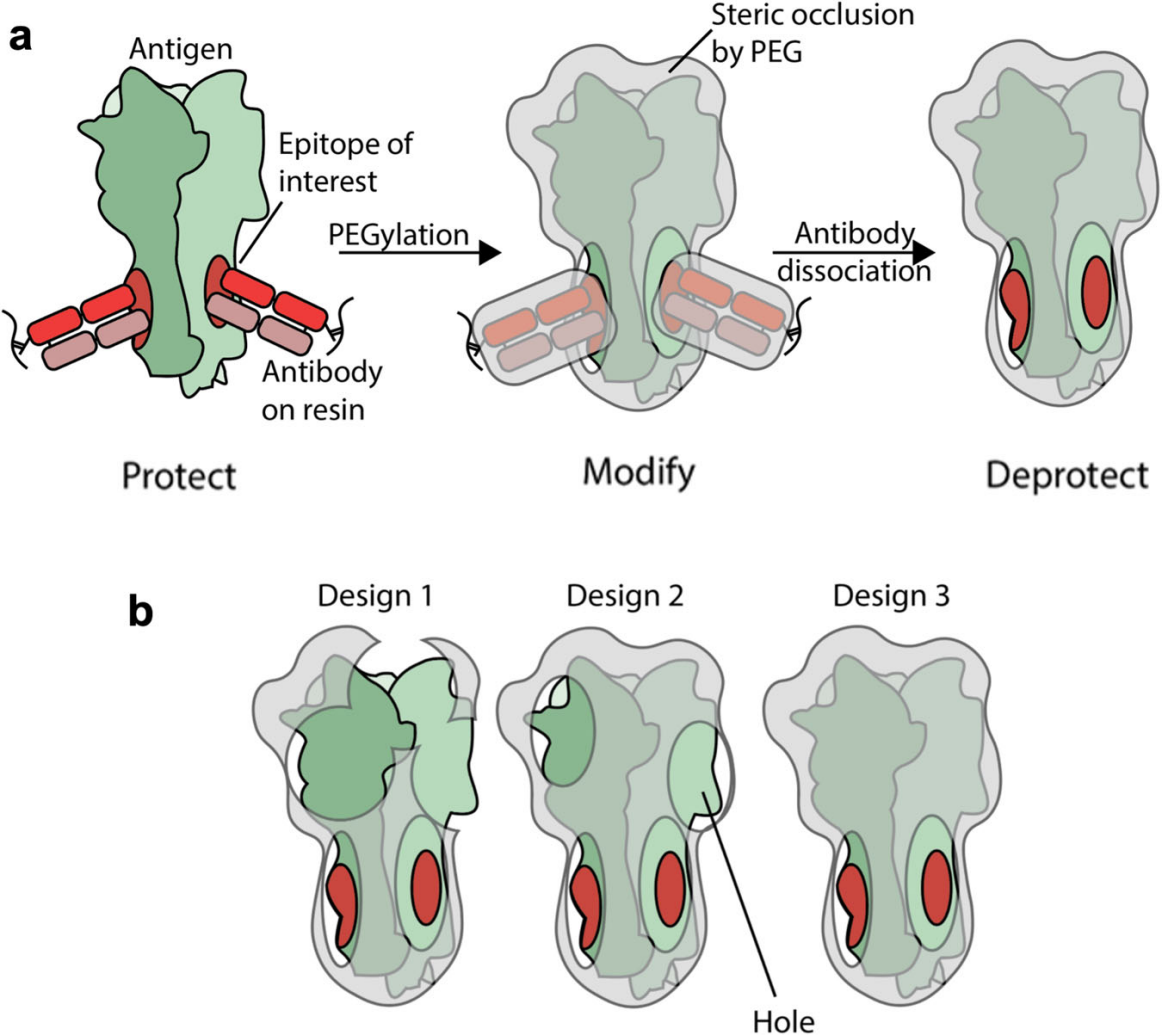
Protect, modify, deprotect (PMD). **a** A general schematic of the PMD strategy. First, the epitope is protected by the target Ab (shown in red). Then the surfaces of the protein complex are modified by PEG to render them non-immunogenic (shown in grey). Finally, the epitope is deprotected by the removal of the Ab. **b** Iterative antigen engineering is required to successively increase the resolution of epitope masking. Holes represent insufficiently masked regions of the antigen and can be characterized using binding experiments with a panel of known off-target antibodies.

Inspired by this use of PEG in drug design and a proof-of-concept of immunosilencing in peptide vaccines^95^, our lab developed an immunofocusing method termed protect, modify, deprotect (PMD) (Fig. 4)^135^. PMD is simply applied– first, the target epitope is protected by a mAb and next, amino acids with functional handles outside of the antibody-antigen interface are covalently modified with short PEG chains. Finally, the mAb is dissociated, thereby exposing the target-epitope. Because PMD immunogens are typically full-length proteins, and made in the presence of the target antibody, the conformational integrity of the epitope is retained.

Our initial test case utilized a stem-directed antibody as the protecting antibody to produce PMD-HA, which retained binding of stem-directed antibodies, but not head-directed antibodies, as intended. Immunization with PMD-HA promoted a more cross-reactive, stem-directed response. We did, however, identify a ‘hole’ in our epitope mask—a region, outside of the epitope of interest, to which antibodies could still bind (Fig. 4b), highlighting the need to evaluate the binding properties of epitope-masked immunogens with panels of off-target antibodies.

A common occurrence and key consideration in epitope masking is the overall decreased immunogenicity of the immunogens—reducing the immunogenicity of off-target epitopes, can also decrease the immunostimulatory potential of the entire protein, resulting in lower antibody titers. Thus, further optimization may be required to increase the overall immunogenicity of these masked proteins, via adjuvants or other excipients^136^. Additionally, applications of epitope masking may be most effective for antigens where the off-target epitopes may induce a detrimental immune response.

## Synergy between Immunfocusing methods

Most immunofocusing methods in this review are orthogonal to one another—meaning they can be applied sequentially or simultaneously to further improve resolution. A good example is the hyperglycosylation of the scaffolded immunogen eOD-GT8 that accurately displays the CD4bs, but elicits anti-scaffold Abs. This has been mitigated with epitope masking in the form of hyperglycosylation of the scaffold at immunogenic regions^137^. Similarly, anti-scaffold immunity could be avoided through a combination of scaffolding and resurfacing. For instance, scaffolded gp120 was resurfaced by mutating 50 residues outside of the 4E10 footprint, which improved thermostability and maintained Ab binding^110^. Mosaic display and protein dissection have also been combined in recent work by Cohen et al. to produce nanoparticles displaying RBDs from eight different zoonotic coronavirus proteins^138^. Immunization of the mosaic produced broader anti-coronavirus responses compared to the WT homosubtypic nanoparticle.

While these represent a subset of the previously explored synergies, we hypothesize that there can be many more. For example, combining hyperglycosylation and PMD could be a promising method to mask more epitope “holes.” Alternatively, antigen reorientation of scaffolded immunogens may better display the target epitope and mitigate the elicitation of off-target epitopes. We envision that combinations of immunofocusing methods can quickly improve neutralization breadth.

## Conclusions

While each of these five methods has different advantages and complexities, there are a number of universal considerations when applying immunofocusing methods. Immunofocusing relies upon proper conformational display of the target epitope in its native state. Target antibody binding to an immunogen is not sufficient to demonstrate that the bound state has the same conformation as the free antigen because the antibody may pull the distorted epitope into the native conformation. Additionally, for viruses where it is particularly difficult to develop bnAbs, high resolution immunofocusing and careful iterative development using a panel of antibodies with known epitopes may be required. During this process, however, it is important to avoid the creation of novel neoepitopes or scaffold immunity. These considerations will be important for the development of novel, effective immunofocusing vaccines.

The choice of immunofocusing methods will depend upon the specific antigen. For instance, low-resolution focusing may be sufficient for HA due to the clear delineation between the immunodominant head and conserved stem domains. Epitope scaffolding is more straightforward for simple linear epitopes such as the helix-turn-helix motavizumab epitope of RSV, rather than complex conformational epitopes such as the VRC01 epitope of HIV-1. In general, while immunofocusing with the highest possible resolution is desirable, this must be weighed against practical challenges such as the overall reduced immunostimulatory potential of the immunogen, and perturbations to the epitope structure which especially accompany methods such as protein dissection and epitope scaffolding. Considerations about which method or methods to apply in which context will also be critical to avoid off-target responses. Advances in computational methods to predict viral escape and mimic epitopes using de novo protein design will also continue to enhance immunofocused vaccine development^139^.

The SARS-CoV-2 pandemic has made clear the necessity to develop universal vaccines against potential pandemic viruses and their variants^140,141^. Immunofocusing against potential pandemic viruses may be a way to mitigate future risks. Indeed, it is likely that we will require significantly higher resolution immunofocusing—perhaps that of a specific antibody epitope(s)—in order to provide broad protection and prepare for future pandemics. The improvement of current methods, and their synergistic applications, as well as the development of novel methodologies, have made the prospect of high-resolution immunofocusing more tractable than ever.
